# The developmental onset of symbolic approximation: beyond nonsymbolic representations, the language of numbers matters

**DOI:** 10.3389/fpsyg.2015.00487

**Published:** 2015-04-29

**Authors:** Iro Xenidou-Dervou, Camilla Gilmore, Menno van der Schoot, Ernest C. D. M. van Lieshout

**Affiliations:** ^1^Department of Educational Neuroscience and LEARN! Research Institute for Learning and Education, Faculty of Psychology and Education, VU University AmsterdamAmsterdam, Netherlands; ^2^Mathematics Education Centre, Loughborough UniversityLoughborough, UK

**Keywords:** numerical cognition, language, nonsymbolic approximate arithmetic, symbolic approximate arithmetic, kindergarten children, number naming system, symbolic arithmetic development, cross-cultural comparison

## Abstract

Symbolic (i.e., with Arabic numerals) approximate arithmetic with large numerosities is an important predictor of mathematics. It was previously evidenced to onset before formal schooling at the kindergarten age (Gilmore et al., 2007) and was assumed to map onto pre-existing nonsymbolic (i.e., abstract magnitudes) representations. With a longitudinal study (Experiment 1), we show, for the first time, that nonsymbolic and symbolic arithmetic demonstrate different developmental trajectories. In contrast to Gilmore et al.’s (2007) findings, Experiment 1 showed that symbolic arithmetic onsets in grade 1, with the start of formal schooling, not earlier. Gilmore et al. (2007) had examined English-speaking children, whereas we assessed a large Dutch-speaking sample. The Dutch language for numbers can be cognitively more demanding, for example, due to the inversion property in numbers above 20. Thus, for instance, the number 48 is named in Dutch “achtenveertig” (eight and forty) instead of “forty eight.” To examine the effect of the language of numbers, we conducted a cross-cultural study with English- and Dutch-speaking children that had similar SES and math achievement skills (Experiment 2). Results demonstrated that Dutch-speaking kindergarteners lagged behind English-speaking children in symbolic arithmetic, not nonsymbolic and demonstrated a working memory overload in symbolic arithmetic, not nonsymbolic. Also, we show for the first time that the ability to name two-digit numbers highly correlates with symbolic approximate arithmetic not nonsymbolic. Our experiments empirically demonstrate that the symbolic number system is modulated more by development and education than the nonsymbolic system. Also, in contrast to the nonsymbolic system, the symbolic system is modulated by language.

## Introduction

Humans and animals seem to be born with an ability to estimate and manipulate abstract magnitudes, namely, nonsymbolic quantities (Flombaum et al., 2005; McCrink and Wynn, 2007; Cantlon, 2012; Starr et al., 2013; for reviews Dehaene et al., 1998; Feigenson et al., 2004; Dehaene, 2011). This ability has been attributed to the so-called approximate number system (ANS), a cognitive system where nonsymbolic numerosities are assumed to be represented and manipulated (Feigenson et al., 2004; Dehaene, 2011). It is a universal system, which is not affected by cross-cultural differences (Pica et al., 2004). In humans, the precision of the ANS increases with age (Halberda and Feigenson, 2008). But, as humans, we also develop higher-order mathematical abilities, based on the use of arbitrary symbols for representing quantities, for example, Arabic notations. In contrast to abstract nonsymbolic representations, symbolic notations allow us to represent quantities precisely. The ANS is often assumed to be linked with the development of our symbolic mathematical abilities (for a review see Feigenson et al., 2013; but see also the review by De Smedt et al., 2013). Symbolic arithmetic processing with large numerosities in an approximate manner has been demonstrated to onset at the age of 5, before the start of formal schooling (Gilmore et al., 2007) and is often assumed to directly map onto one’s readily accessible nonsymbolic representations (Lipton and Spelke, 2005; Gilmore et al., 2007; Mundy and Gilmore, 2009). But is this developmental onset of symbolic arithmetic processing universal? Symbols carry with them their phonological representations, which in turn depend on the language one uses (Carey, 2004; Pica et al., 2004). Thus, even though Arabic symbols are used widely, the way they are named varies significantly across different languages (e.g., Pica et al., 2004; Dehaene, 2011). Early symbolic processing skills have been consistently proven to be significant predictors of math achievement (for a review see De Smedt et al., 2013; see also Göbel et al., 2014b; Lyons et al., 2014), even beyond general processing skills, such as working memory (WM) abilities (Xenidou-Dervou et al., 2013). Therefore, a better understanding of their developmental onset and factors affecting them is rendered necessary. This manuscript investigates, for the first time, the developmental trajectories of nonsymbolic and symbolic arithmetic skills and the roles that development, education and language play in this process.

We often find ourselves in a hurry looking at price tags and making a quick estimation such as: “This package costs 38 euros plus 17 for the extras; that’s more than the 50 euros I have with me!” Gilmore et al. (2007) demonstrated that the ability to perform such type of symbolic arithmetic with large numerosities starts at the age of 5, namely before starting primary school instruction. Five years-old children could perform well above chance level on symbolic arithmetic problems, which entailed numbers from 5 to 58. These problems asked for abilities that enable one to give an approximate response, otherwise known as approximation skills (Xenidou-Dervou et al., 2013). Gilmore et al.’s (2007) findings were surprising: this study suggested that children are capable of a form of symbolic arithmetic without needing formal schooling. Of course, the question that rose was how could such young children solve these problems? An explanation was derived from the finding that performance on this type of symbolic arithmetic problems demonstrated exactly the same signature effects as those appearing in corresponding ANS measures, namely in the nonsymbolic versions (Gilmore et al., 2007). It is often assumed that the ANS influences the symbolic number system (Feigenson et al., 2013) and that symbolic representations directly map onto readily accessible ANS representations (Lipton and Spelke, 2005; Mundy and Gilmore, 2009). The primary signature effect of approximation skills (nonsymbolic or symbolic), is the well-known ratio effect: the more the ratio between two quantities or symbols deviates from 1, the easier it is to compare them (Pica et al., 2004; Barth et al., 2005, 2006; Gilmore et al., 2007, 2010; Xenidou-Dervou et al., 2013, 2014). This is based on the assumption that we perceive numerosities on the basis of a mental number line (Izard and Dehaene, 2008). The further two quantities are from each other, the less their representational overlap on this mental number line and thus the easier it is to compare them. It has also been shown that approximate comparison performance is similar to approximate addition performance (Gilmore et al., 2007).

Since Gilmore et al.’s (2007) study, few have examined the corresponding arithmetic processing skills in such young children. Xenidou-Dervou et al. (2013) assessed kindergarteners’ nonsymbolic and symbolic approximation skills in addition and comparison. Using structural equation modeling, Xenidou-Dervou et al. (2013) demonstrated that at the kindergarten stage nonsymbolic approximate addition and comparison load on a single nonsymbolic approximation latent factor, whereas symbolic approximate addition, and comparison load on an distinct factor, that of symbolic approximation. In this study, 5 years-old children performed above chance in all nonsymbolic and symbolic approximation tasks without resorting to known alternative systematic response strategies. They also demonstrated the characteristic ratio effect in all approximation tasks with the exception of one: kindergarteners performance in the symbolic approximate addition task did not demonstrate the ratio effect. Performance in this task was relatively low and close to chance level (56.53%) indicating that the children had difficulties with this task. Furthermore, the authors demonstrated that even though nonsymbolic and symbolic arithmetic processing were related in kindergarten age, they were two distinct abilities (Xenidou-Dervou et al., 2013). These findings provided further proof that symbolic arithmetic, as a linguistically mediated system, does not necessarily map only onto nonsymbolic processing at the kindergarten age (see also Sasanguie et al., 2014).

The fact that kindergarteners performed poorly in symbolic approximate addition in Xenidou-Dervou et al.’s (2013) study and demonstrated no ratio effect contradicted Gilmore et al.’s (2007) findings. Xenidou-Dervou et al. (2013) claimed that this difference might be attributed to task or sample characteristic differences. The symbolic approximate arithmetic tasks used in Gilmore et al. (2007) and Xenidou-Dervou et al.’s (2013) studies differed on certain *task-design characteristics*. The latter entailed a larger range of numerosities (6–70) and the numbers were not read aloud to the children. They merely saw the displayed symbols. These characteristics could have made the task harder and thus might have not captured the onset of the skill in question. Or perhaps the task’s design failed to capture the desired ability in general; if that were the case, then one would not expect a ratio effect to appear in grade 1 either. An alternative explanation though could be that the large sample in Xenidou-Dervou et al. (2013) did not have adequate symbolic knowledge to be able to successfully solve these symbolic arithmetic problems even if they only asked for an approximate response. This would imply that with time and instruction – and thus the gradual automatization of symbols, children’s performance would improve. In other words, the onset of symbolic approximate arithmetic would be expected to take place in grade 1.

Previous studies have shown that precision in nonsymbolic and symbolic magnitude comparison increases with age (Halberda and Feigenson, 2008; Holloway and Ansari, 2009). However, to our knowledge, no previous study has compared the developmental trajectories of nonsymbolic and symbolic *arithmetic* processing. Since symbolic processing necessitates additional cognitive processes related to symbolic knowledge beyond the simple underlying ANS representations, we expected nonsymbolic and symbolic approximate arithmetic to demonstrate different developmental trajectories. As children enter grade 1, they receive formal school instruction and thus acquire symbolic knowledge. Therefore, we hypothesized that symbolic arithmetic would show greater developmental increase compared to the corresponding nonsymbolic arithmetic processing skills. Whereas the characteristic ratio effect in nonsymbolic approximation would be evident across both kindergarten and grade 1, we expected that in symbolic approximate addition it would become significant only after the start of formal schooling, namely in grade 1.

## Experiment 1

### Method

#### Participants

This experiment was part of a large-scale longitudinal project, known as the MathChild project^1^. The project started with 444 kindergarteners (*M*_age_ = 5.59 years, *SD* = 0.35) from 25 schools around the Netherlands (for more information, including SES information, please see Participants in Xenidou-Dervou et al., 2013). A year later – in grade 1 – 396 of these children were tested again on the tasks presented in this study. Dropouts were primarily due to changing schools. All dropouts were excluded from the analyses. In grade 1 (*M*_age_ = 6.50 years, *SD* = 0.32), the sample consisted of 221 boys and 175 girls. All children spoke Dutch and 95.96% of them had Dutch nationality. Legal guardians’ written consents were received for all children.

#### Procedure

All children were tested individually in quiet settings within the school facilities by trained experimenters, who used a detailed protocol with written instructions. The data reported in this study regard a subset of tasks from the MathChild project. At both time points (kindergarten and grade 1), testing started in November and ended in January of the given academic year. In grade 1, testing included two sessions. The tasks reported in the present study were part of the second session. The order of presentation of the tasks was controlled for by alternating the order of the tasks. Children received small tokens after each session for encouragement. Kindergarten data have been previously reported in Xenidou-Dervou et al. (2013).

#### Materials

Tasks used were computerized and presented in E-prime version 1.2 (Psychological Software Tools, Pittsburgh, PA, USA) with HP Probook 6550b type laptops.

##### Nonsymbolic approximate addition

**FIGURE 1:**
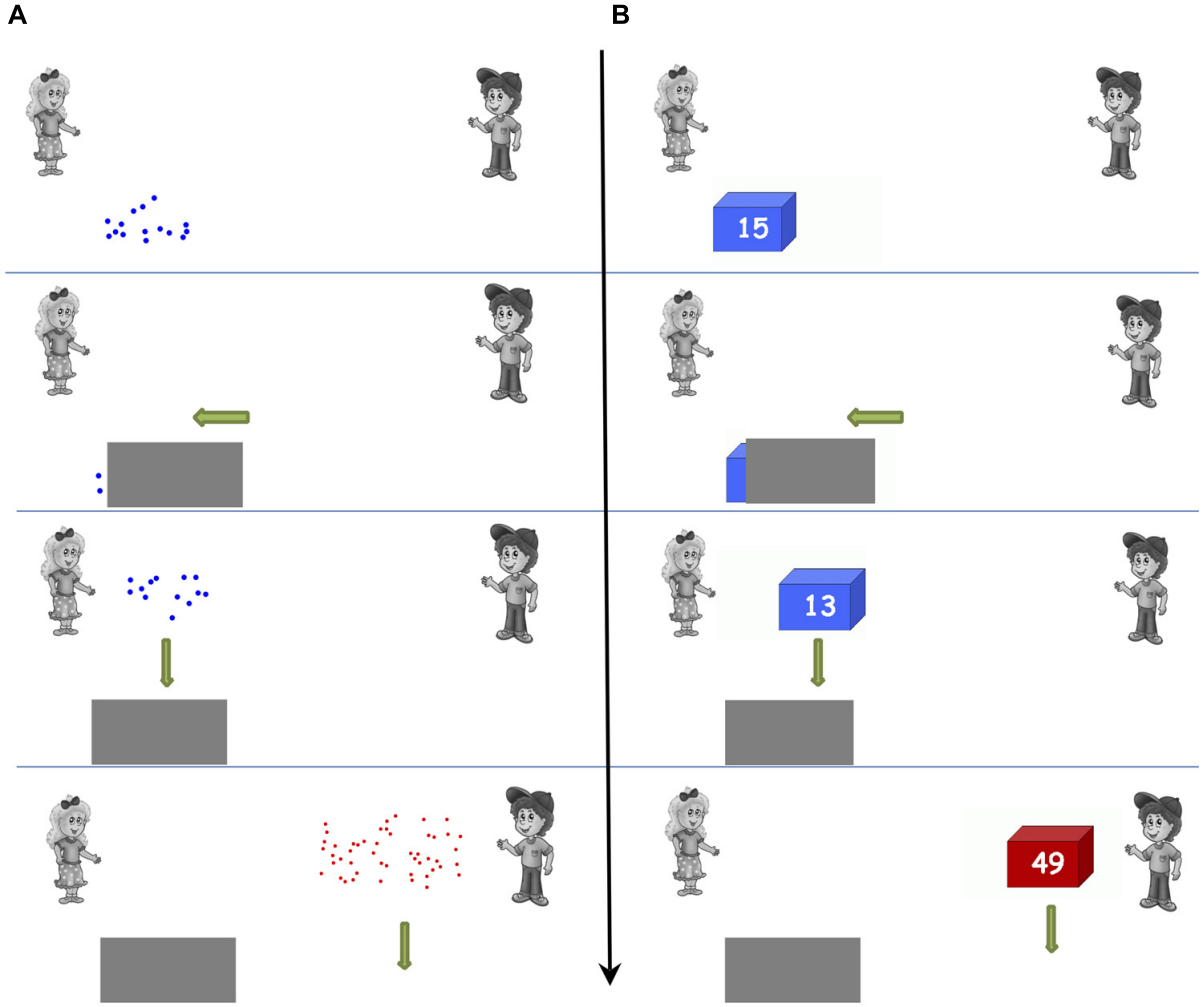
**Nonsymbolic **(A)** and symbolic **(B)** approximate addition example trials**.

Children saw an image of a girl (Sarah) and a boy (Peter) on the far top left side and right side of the screen correspondingly. A trial entailed the following sequence of steps (see **Figure 1A**): (1) Sarah got an amount of blue dots, (2) These were covered up by a gray box, (3) Then she got some more blue dots, (4) These were now all behind the gray box, (5) Lastly, Peter got some red dots. The question they had to answer was: “Who got more dots?” Participants were instructed to press the blue response box in front of them, if they thought Sarah received more dots, or the red response box, if they thought Peter received more dots. Each animated event lasted 1300 ms and between them there was a 1200 ms interval. Children were instructed to respond as correctly and as fast as possible. Once the red dots appeared on the screen, the children had a maximum of 7000 ms to respond. If they did not respond on time, the trial was automatically coded as incorrect. The fast interchange of events and response process prevented children from counting the dots. Between trials, there was an interval of 300 ms.

Numerosities in this task ranged from 6 to 70. The sum of the blue addends differed with the comparison red addend by three ratios with eight trials in each ratio level: 4/7 (easy ratio), 4/6, (middle), and 4/5 (difficult). Similar to previous studies (Barth et al., 2006, 2008; Gilmore et al., 2010; Xenidou-Dervou et al., 2013, 2014), trials were constructed in a manner that allowed the *post hoc* examination of the use of possible alternative systematic response strategies not related to approximate addition, for example, if children only pressed the red or blue button without adding and comparing the addends (see Appendices in Gilmore et al., 2010; Xenidou-Dervou et al., 2013, 2014). Dots were constructed in MATLAB 7.5 R2007b. As in previous studies, to avoid children’s responses relying on the physical features of the dots, we controlled for dot size, total surface area, total contour length, and density (Barth et al., 2006; Gilmore et al., 2010; Xenidou-Dervou et al., 2013, 2014).

In kindergarten, children received six practice trials in order to optimally comprehend the task (see Barth et al., 2005, 2006; Xenidou-Dervou et al., 2013). In grade 1, they received two practice trials to recall the task’s demands. The task included 24 test trials (see Supplementary Material). No feedback was provided during testing aside from occasional verbal encouragement when necessary.

##### Symbolic approximate addition

As in previous studies (e.g., Xenidou-Dervou et al., 2013), this task was identical to its nonsymbolic version with the key difference that the dots were now replaced with blue or red boxes displaying the corresponding Arabic notation (see **Figure 1B**). Children were asked to provide an approximate response, namely they were asked to respond as correctly and as fast as possible to the question “Who got more stickers?” The child was asked to estimate, which was more: the sum of the blue number of stickers or the red. The fast interchange of the sequential events and the fact that a response had to be produced within 7000 ms maximum encouraged an approximate response.

#### Results

Children performed above chance level (50%) in all tasks in kindergarten: nonsymbolic addition [*M* = 63.56%, *SD* = 10.81, *t*(392) = 24.88, *p* < 0.001], symbolic addition [*M* = 57.06%, *SD* = 11.88, *t*(392) = 11.78, *p* < 0.001] and grade 1: nonsymbolic addition [*M* = 67.76%, *SD* = 14.19, *t*(395) = 33.12, *p* < 0.001], symbolic addition [*M* = 67.07%, *SD* = 10.81, *t*(395) = 23.93, *p* < 0.001]. Correlations between the assessed measures are presented in **Table 1**.

**Table 1:** Correlations between the nonsymbolic and symbolic arithmetic measures assessed in kindergarten and grade 1.

	1	2	3
(1) Kindergarten nonsymbolic addition			
(2) Kindergarten symbolic addition	0.24^∗∗∗^ (393)		
(3) Grade 1 nonsymbolic addition	0.19^∗∗∗^ (394)	0.13^∗∗^ (394)	
(4) Grade 1 symbolic addition	0.17^∗∗∗^ (394)	0.41^∗∗∗^ (394)	0.27^∗∗∗^ (396)

Parentheses include the *N* sample within the specific analysis. ^∗∗^*p* ≤ 0.01; ^∗∗∗^*p* ≤ 0.001.

**FIGURE 2:**
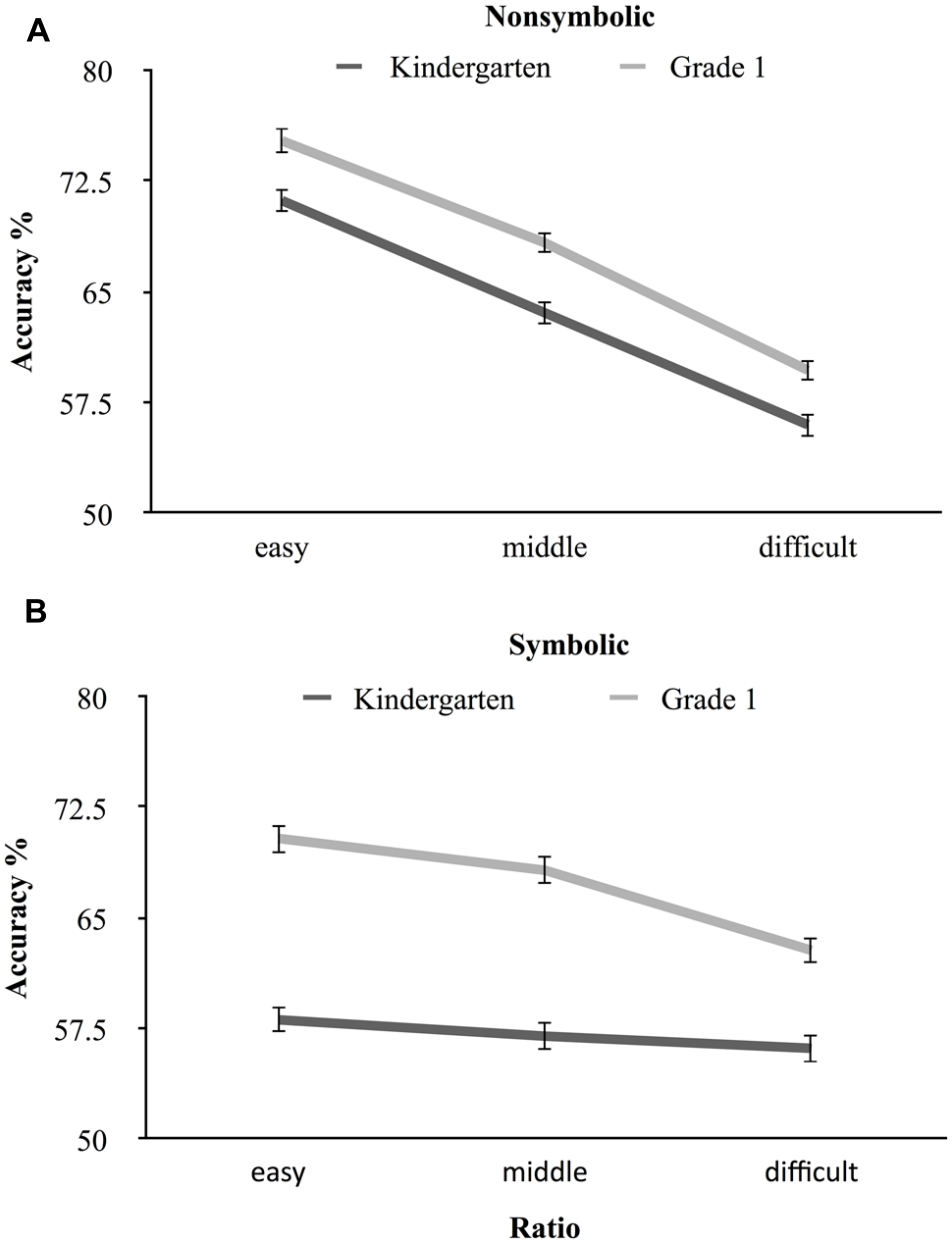
**The developmental trajectories of nonsymbolic **(A)** and symbolic **(B)** approximate addition in kindergarten and grade 1.** In the symbolic condition, the ratio effect became significant in grade 1.

To compare the developmental trajectories of nonsymbolic and symbolic approximate addition, we conducted a 2 (Task: nonsymbolic and symbolic) × 3 (Ratio: easy, middle, difficult) × 2 (Year: kindergarten and grade 1) repeated measures ANOVA. Mauchly’s test indicated that the assumption of sphericity had been violated for Ratio, χ^2^(2) = 8.99, *p* = 0.011, and the Task by Ratio by Year interaction, χ^2^(2) = 12.39 *p* = 0.002. Therefore, we corrected the degrees of freedom using Greenhouse–Geisser estimates. Results demonstrated main effects of Task, *F*(1,392) = 37.33, *p* < 0.001, $\eta_{p}^{2}$ = 0.09, Ratio, *F*(1.96,766.58) = 192.02, *p* < 0.001, $\eta_{p}^{2}$ = 0.33, Year, *F*(1,392) = 178.72, *p* < 0.001, $\eta_{p}^{2}$ = 0.31, and the expected significant interaction effect of Task by Ratio by Year, *F*(1.94,760.29) = 3.41, *p* = 0.035, $\eta_{p}^{2}$ = 0.01 (see **Figure 2**). To examine the simple effects two additional analyses were conducted for each task (nonsymbolic and symbolic) separately. For nonsymbolic addition, we found significant main effects of Year, *F*(1,393) = 36.99, *p* < 0.001, $\eta_{p}^{2}$ = 0.09, and Ratio, *F*(1.97,774.01) = 234.34, *p* < 0.001, $\eta_{p}^{2}$ = 0.37 but not their interaction. For symbolic addition, results showed significant main effects of Year, *F*(1,393) = 196.49, *p* < 0.001, $\eta_{p}^{2}$ = 0.33, and Ratio, *F*(2,392) = 18.47, *p* < 0.001, $\eta_{p}^{2}$ = 0.09, but for this task their interaction was also significant, *F*(1.95,767.78) = 7.29, *p* = 0.001, $\eta_{p}^{2}$ = 0.02. For this interaction, further simple effect analyses demonstrated that, as expected, in the symbolic condition the ratio effect was only significant in grade 1, *F*(2,394) = 25.17, *p* < 0.001, $\eta_{p}^{2}$ = 0.11, and not in kindergarten, *F*(1.95,764.55) = 1.42, *p* = 0.244, $\eta_{p}^{2}$ = 0.00. Thus, as hypothesized, nonsymbolic and symbolic approximate arithmetic processing demonstrated different ratio effect developmental trajectories. The ratio effect in symbolic approximate addition became significant in grade 1, **Figure 2B**.

#### Discussion

Experiment 1 confirmed our hypothesis that nonsymbolic and symbolic arithmetic processing demonstrate different developmental trajectories. Nonsymbolic acuity increased steadily across time (Halberda and Feigenson, 2008), however, symbolic processing showed a larger increase with the start of formal schooling in grade 1. In other words, symbolic arithmetic processing seemed to be modulated by age and education more than nonsymbolic arithmetic processing. This result also indicated that the symbolic approximate arithmetic task did indeed tap the ability in question: we found a significant ratio effect in symbolic approximate addition in grade 1. So, in this large Dutch sample symbolic approximate arithmetic appeared to onset in grade 1, when school instruction had started. However, the question remained: why was performance in the symbolic approximate addition task so low at the kindergarten level? Based on Gilmore et al.’s (2007) results, the skill to conduct computations with large symbolic quantities in an approximate manner should start already at the age of 5 years. As described earlier, the difference in results with Gilmore et al. (2007) could still be due to task-design differences. However, there is another, striking difference between the present study and Gilmore et al.’s (2007) study. In Experiment 1, we examined Dutch-speaking children, whereas Gilmore et al. (2007) examined English-speaking children.

There is compelling evidence across interdisciplinary literature demonstrating the importance of the ability to effectively add and compare symbols for children’s mathematical achievement (for a review see De Smedt et al., 2013). Given the significant role that symbolic approximation plays in kindergarten math achievement (Xenidou-Dervou et al., 2013), it is imperative to identify the language related factors that play a role in the developmental onset of these skills.

## Experiment 2

So far, the characteristic ratio effect in approximation tasks has been considered universal even when symbols (Arabic numerals) are used. However, the level of transparency of a number naming system has been demonstrated to influence performance even in non-verbal symbolic tasks where the Arabic notation is merely shown, not heard (Nuerk et al., 2005; Helmreich et al., 2011; Göbel et al., 2014a). For example, an essential difference in naming numbers in English versus Dutch (as well as German and other, see Comrie, 2005) is the fact that the latter entail the so-called inversion property. In English, two-digit numbers above 20, such as the number 48, are named in the same order as they are written: first the decades and then the units. In Dutch, however, it is the opposite: first, one names the units and then the decades. So, the number “48” is actually named “eight and forty” (in Dutch: “acht en veertig”). The inversion property has been reported to negatively affect children’s symbolic numerical processing. Specifically, Göbel et al. (2014a) demonstrated that it hinders German-speaking (inversion language) second graders’ complex two-digit symbolic addition versus their Italian-speaking peers. Furthermore, Helmreich et al. (2011) found German-speaking first graders’ number line skills to be less accurate compared to their Italian-speaking peers. Therefore, it could be expected that Dutch-speaking children, similar to German-speaking children would have a disadvantage in their symbolic numerical processing with large numbers due to the demanding Dutch number naming system. Symbolic approximate arithmetic tasks such as those used in Gilmore et al.’s (2007) and the present study entail many two-digit numerosities across their trials and the response on these trials cannot be made by just judging on the basis of the decade of a two-digit number (see Supplementary Material).

Let us consider the cognitive process that could occur when estimating a symbolic number above 20 in English and in Dutch. In English, the phonological representation of an Arabic two-digit number could involve the following two steps: the child (silently) can vocalize the decades, which he/she then can approximately position on an assumed mental number line. Then, the child can vocalize the units with which he or she fine-tunes approximately the position on the mental number line. In Dutch, the corresponding process appears more demanding. The child first can (silently) vocalize the units but this step would not allow him/her to make an approximate decision on the entire number’s position on a mental number line. Instead, this action must be delayed till after the child has vocalized the decades. Meanwhile, the child has to retain the units in his/her WM. In other words, the number naming process in Dutch appears to require more cognitive steps, which will occupy more WM resources. As described earlier, the ratio effect in approximation is assumed to occur because we estimate on the basis of a mental number line where numerosities that are closer to each other have a larger representational overlap and are thus harder to compare. Therefore, the lack of a ratio effect in Dutch kindergarteners’ symbolic approximate arithmetic could be due to their demanding number naming system, which would manifest itself as a WM overload. Previous studies have shown that WM is highly related to children’s inversion errors when transcoding, namely writing 48 when hearing “forty eight,” in German or Czech (Zuber et al., 2009; Pixner et al., 2011). In particular, these studies found that the Central Executive (CE) component of WM, on the basis of to the multicomponent model of WM (Baddeley and Hitch, 1974; Baddeley, 2012), was the most predictive component of inversion-related errors. To our knowledge, the role of WM in symbolic approximate addition in an inversion number naming system such as the Dutch has not been previously addressed.

Cross-cultural studies on numerical skills, thus far, have been conducted with primary school children. Early numeracy skills, however, have been shown to play a role in children’s math achievement already from the kindergarten age (e.g., Booth and Siegler, 2006; LeFevre et al., 2010; Mazzocco et al., 2011; Geary et al., 2013; Xenidou-Dervou et al., 2013; Bartelet et al., 2014; Hornung et al., 2014). Furthermore, previous cross-cultural studies did not account for the children’s nonsymbolic skills. It could be argued that the groups compared may differ on the basis of their general ability to estimate magnitudes, namely their ANS, not symbolic notations *per se*. We hypothesized that sample differences on the basis of the number naming system children use significantly affects symbolic arithmetic processing beyond their ANS skills. Drawing on the aforementioned assumptions, three clear predictions could be made: (1) Dutch-speaking kindergarteners would have similar ANS skills with matched English-speaking children but would demonstrate a disadvantage in symbolic approximate arithmetic. (2) Dutch-speaking kindergarteners would demonstrate a WM overload in symbolic approximate arithmetic, but not nonsymbolic. (3) The ability to name two-digit numbers would only correlate with symbolic approximate processing, not nonsymbolic. In order to address these hypotheses, we extended our study with a second experiment in which data was collected from an English-speaking comparison group.

### Method

#### Participants

In addition to the existing kindergarten Dutch sample, we tested 54 English-speaking children in the UK (*M*_age_ = 5.33 years, *SD* = 0.49; 28 boys). Children, who spoke a second language that entailed the inversion property in their number naming system (*n* = 2) and those with missing data were excluded (*n* = 10). We aimed at having two samples (English-speaking and Dutch-speaking) that had comparable educational and SES backgrounds in order to effectively examine their differences on the basis of language.

With respect to SES, McNeil et al. (2011) have shown that it can influence preschoolers’ approximate addition skills. In the present study, SES background was indicated by the parents’ level of education. Preliminary analyses in the Dutch sample (used in Experiment 1) had shown that fathers’ level of education significantly correlated with their children’s symbolic approximate addition (*r* = 0.10, *p* = 0.045). Mothers’ level of education did not correlate with the approximation measures. The large Dutch-speaking sample’s fathers came from variable SES backgrounds (Xenidou-Dervou et al., 2013). The relatively smaller English-speaking sample, however, consisted of children whose fathers had received higher levels of education. Thus, to control for SES differences across the two samples (UK and NL), children from the NL sample with fathers who had received low educational levels [below HAVO (Dutch educational system)] were excluded from the analyses. The comparison of the two countries’ educational systems was based on the official education module comparison developed by the Nuffic (2013), which resulted in seven educational levels. On the basis of these exclusion criteria, the two samples’ fathers’ SES no longer differed, *t*(47.81) = 0.18, *p* = 0.811 (NL: *M* = 5.93, *SD* = 0.83, UK: *M* = 5.91, *SD* = 0.93).

It was also important that the two samples (UK and NL) had similar educational background. The Dutch kindergarten sample (see Experiment 1) had not received any formal math instruction. Formal instruction in the NL starts in the third year of schooling (“groep 3”). In the UK, however, formal math instruction starts earlier. Therefore, we purposefully assessed younger children in the UK, who had also not yet received formal math instruction. Below the resulting samples from the two countries are described.

The Dutch-speaking sample used in this experiment’s analyses consisted of 204 children (*M*_age_ = 5.58 years, *SD* = 0.35; 115 boys), 98.04% had Dutch nationality. All children spoke Dutch. According to teacher reports 173 of these children did not speak a second language, for 31 of these children, however, this information was not available as they had moved and changed schools before the time of inquiry. In the Dutch-speaking sample, 92.2% of their fathers and 63.2% of their mothers held an undergraduate or higher academic degree. All the Dutch-speaking children already attended kindergarten (“groep 2” in the Dutch educational system). In this grade in the Netherlands children do not receive structured educational instruction.

The English-speaking sample consisted of 42 children (*M*_age_ = 5.31 years, *SD* = 0.53; 23 boys), 97.62% had a UK nationality. All children spoke English and two of them spoke a non-inversion second language. In this sample, 76.2% of their fathers and 78.6% of their mothers held an undergraduate or higher academic degree. The UK children were tested before the start of the school year during the summer period. At this time the children had only completed 1 year in school. The first year (Reception) is part of the Foundation Stage (age 0–5) during which children learn through play-based activities. In the UK, formal instruction begins during the second year of schooling. As intended, the English-speaking sample was significantly younger compared to the Dutch-speaking sample (*p* = 0.003).

#### Procedure

The English-speaking sample was assessed subsequently to the Dutch-speaking sample. Testing took place during the University of Nottingham’s Summer Scientist Week^2^. This is an annual research and outreach event during which parents and their children visit the university, play games and take part in studies. SES diversity for this event is highly promoted. Parents/legal guardians provided written consent and SES information. The children were tested in two 20-min sessions. After each session they received tokens to sustain their motivation for participation. For information on the procedure followed in the Dutch sample see Experiment 1. Experimenters in both samples used the same instruction and testing protocol.

#### Materials

All the tasks were presented with the same hardware and software as in Experiment 1. The English-speaking sample was assessed on measures that the Dutch sample had been previously tested on (see Xenidou-Dervou et al., 2013). Additionally, the English-speaking sample was also tested on the Naming Large Numbers test.

##### Nonsymbolic and symbolic approximate addition

See Experiment 1 (see Materials). The Supplementary Material demonstrates the trials included in this task. It should be noted that in five of these trials (see Supplementary Material, trial numbers: 12, 13, 17, 21, 24) the naming process of their numbers did not differ across English and Dutch on the basis of the inversion property. Since the trials for this task have been stringently constructed based on several control dimensions (see for example Xenidou-Dervou et al., 2013) and due to the comparison with its nonsymbolic counterpart, we opted to keep these five trials. Nevertheless, all trials in the “easy ratio” included two-digit numbers above 20, which are characterized by the inversion property in the Dutch language and not in the English language. We, therefore, expected the difference between the UK and the NL children to be primarily evident in this ratio.

##### Exact addition

The exact symbolic addition task (see Jenks et al., 2009; Xenidou-Dervou et al., 2013) assesses children’s addition skills in the familiar form of “a + b = c.” It entailed 15 addition problems, where “a” and “b” were larger than 1 and never equal. The first 10 problems were simple (*c* < 10) and the last five were harder (10 < *c* < 16). The child saw each addition problem on the screen and had to give as correctly and as fast as possible a verbal response for the exact number of the sum. This task demonstrates high levels of internal consistency (Xenidou-Dervou et al., 2013).

##### Counting skills

The English and the Dutch version of four subscales from the Early Numeracy Test – Revised (ENT-R, version A) were used to assess children’s counting abilities (Van Luit and Van de Rijt, 2009). The subscales assessed (20 items) focused on the child’s ability to: (1) use number words (counting forward and backward up to maximum 20); (2) execute structured counting (counting while pointing to objects); (3) conduct resultative counting (counting without pointing to objects); (4), and their general understanding of numbers and how to use the counting system in everyday life.

##### Working memory

The English and Dutch versions of two widely known tasks (e.g., Alloway et al., 2004; Xenidou-Dervou et al., 2013) were used to assess children’s WM capacity. We had hypothesized that the Dutch number naming system would be phonologically more demanding than the English one. Therefore, we focused on the phonological loop (PL) of the WM construct and its interaction with CE WM resources (Baddeley, 2002; Repovs and Baddeley, 2006).

The *Word Recall Forward* task taps children’s PL capacity, namely, the ability to retain phonological information. The child heard a series of recorded high frequency unrelated words and had to repeat them in the same order. After four correct recalls, the child was automatically advanced to the next level that entailed one extra word. A response was registered as correct if the child recalled the word(s) correctly and in the same order as heard. The task would discontinue after three incorrect responses within one level of difficulty.

The *Word Recall Backward* task taps children’s CE capacity, specifically the ability to control, regulate and manipulate phonological information. The task’s characteristics were identical to the Word Recall Forward task, only now the child was asked to recall the words he/she heard backwards. This task started with a string of two words.

##### Naming large numbers test

This test assessed children’s ability to name numbers above 20. The children saw a number on the screen, which remained until they gave a verbal response. They were asked to name each number as accurately and quickly as possible. The experimenter pressed a button the moment the child responded, which registered their response time (RT). Nine numbers were used, which are included within the trials of the symbolic approximate arithmetic task and involve the inversion property in the Dutch number naming system but not in the English: 25, 36, 52, 21, 49, 67, 48, 24, and 63 (see Supplementary Material). The order of presentation of the numbers was randomized.

### Results

#### Descriptive Statistics

**Table 2** presents the two groups’ descriptive statistics on the control measures. ANOVAs were conducted to compare performance across the two samples. As expected, they had similar simple addition (in the form of “a + b = c”) and counting skills. However, the Dutch-speaking children had higher WM skills, as they were significantly older than the English-speaking children. We, therefore, controlled for PL and CE WM skills within our subsequent analyses.

**Table 2:** Descriptives and comparisons a cross the two groups.

Tasks	Country	*M*	*SD*	Comparisons
Exact addition	NL	6.22	4.55	ns
	UK	5.12	5.01	
Early numeracy	NL	11.42	4.18	ns
	UK	10.26	4.37	
Phonological loop (PL)	NL	13.29	2.55	*p* < 0.001
	UK	11.64	3.54	
Central executive (CE)	NL	4.65	1.88	*p* = 0.001
	UK	3.55	2.19	

NL, Netherlands; UK, United Kingdom.

#### Approximate Addition Comparisons

**FIGURE 3:**
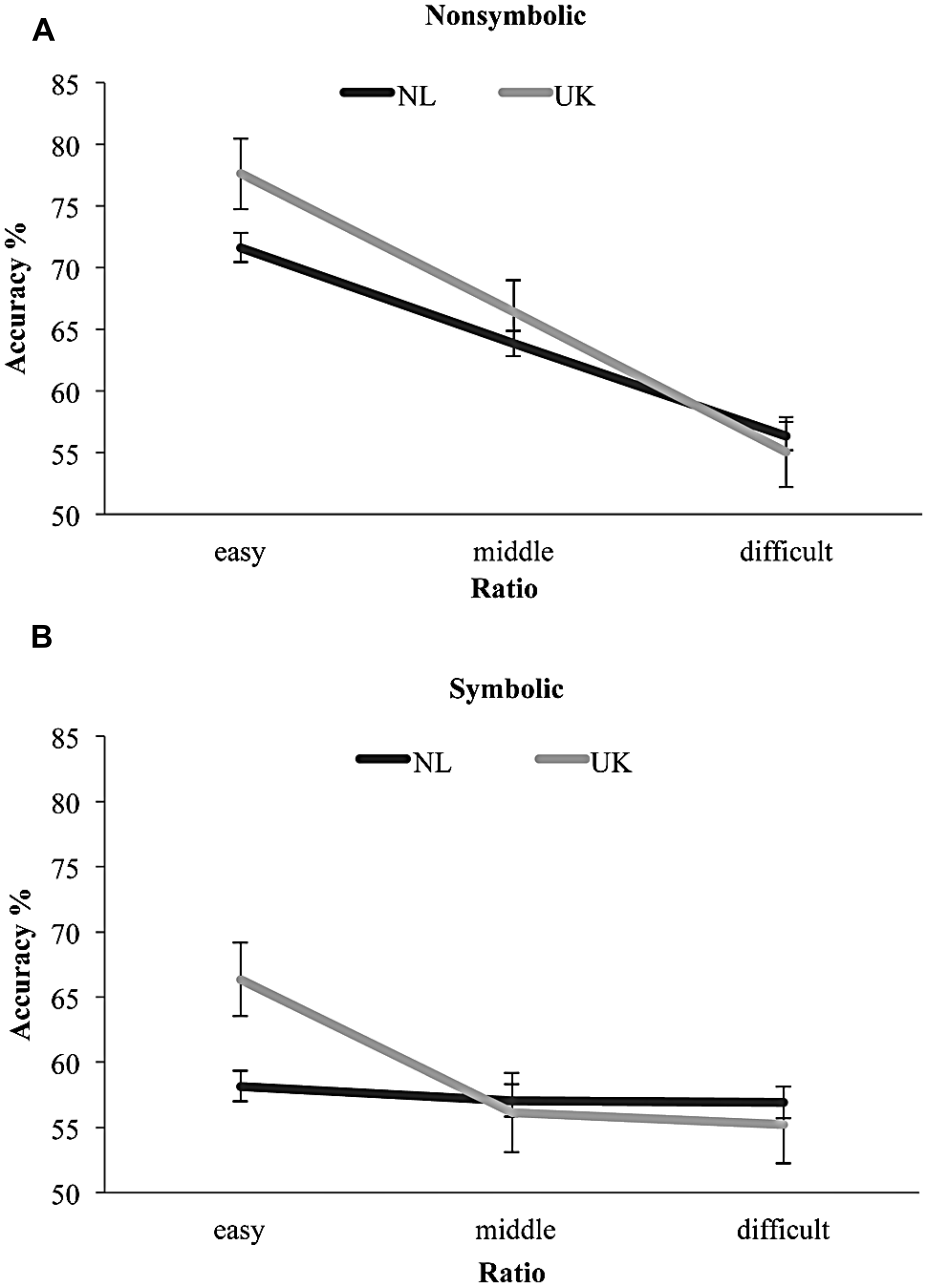
**Dutch-speaking and English-speaking children’s nonsymbolic and symbolic approximate addition ratio performance.** Both samples had similar nonsymbolic approximation skills **(A)** but differed on the easy ratio of symbolic approximate arithmetic **(B)**.

To examine our first and second hypotheses, we conducted a 3 (Ratio: easy, middle, difficult) × 2 (Country: NL and UK) × 2 (Task: nonsymbolic and symbolic) repeated measures ANCOVA with PL and CE performance as centered covariates (see Thomas et al., 2009). Since the sample sizes across the groups were unequal, Type III sum of squares were used (Maxwell and Delaney, 2004). Box’s *M*-test of equality of covariance matrices in all analyses were not significant. As expected, we found a significant Task by Ratio by Country by CE interaction effect, *F*(2,239) = 4.89, *p* = 0.008, $\eta_{p}^{2}$ = 0.04 (see **Figure 3**). In accordance to our hypothesis, CE WM resources appeared to modify the interaction between Task, Country and Ratio. To clarify this 4-way interaction, simple effect analyses were conducted within each task (nonsymbolic and symbolic). For nonsymbolic approximate addition, only the expected main ratio effect was found, *F*(1.89,452.92) = 49.81, *p* < 0.001, $\eta_{p}^{2}$ = 0.17. For the symbolic condition, results demonstrated: a main effect of Ratio, *F*(1.92,460.20) = 6.21, *p* = 0.003, $\eta_{p}^{2}$ = 0.03, a Ratio by Country interaction, *F*(2,239) = 4.73, *p* = 0.010, $\eta_{p}^{2}$ = 0.04, and as expected a Ratio by Country by CE interaction, *F*(2,239) = 5.37, *p* = 0.005, $\eta_{p}^{2}$ = 0.04. Therefore, as hypothesized, the two groups did not differ on the basis of their nonsymbolic approximate skills but only on their symbolic approximate addition performance. Pairwise comparisons indicated that the English-speaking children performed better on the easy ratio of the symbolic approximate addition task (*p* = 0.008), where all trials included an “inversion number.”

To identify the role of the CE component of WM in this interaction, regression equations were constructed with unstandardized regression coefficients on the basis of the parameter estimates derived from the ANCOVA:

$$Y_{easy\,\;ratio}=66.338+4.076X_{CE}-8.177X_{country}-2.939X_{CE}X_{country}$$

$$Y_{middle\,\;ratio}=56.114-0.641X_{CE}+0.931X_{country}+2.494X_{CE}X_{country}$$

$$Y_{difficult\,\;ratio}=55.186+2.575X_{CE}+1.721X_{country}-1.746X_{CE}X_{country}$$

**FIGURE 4:**
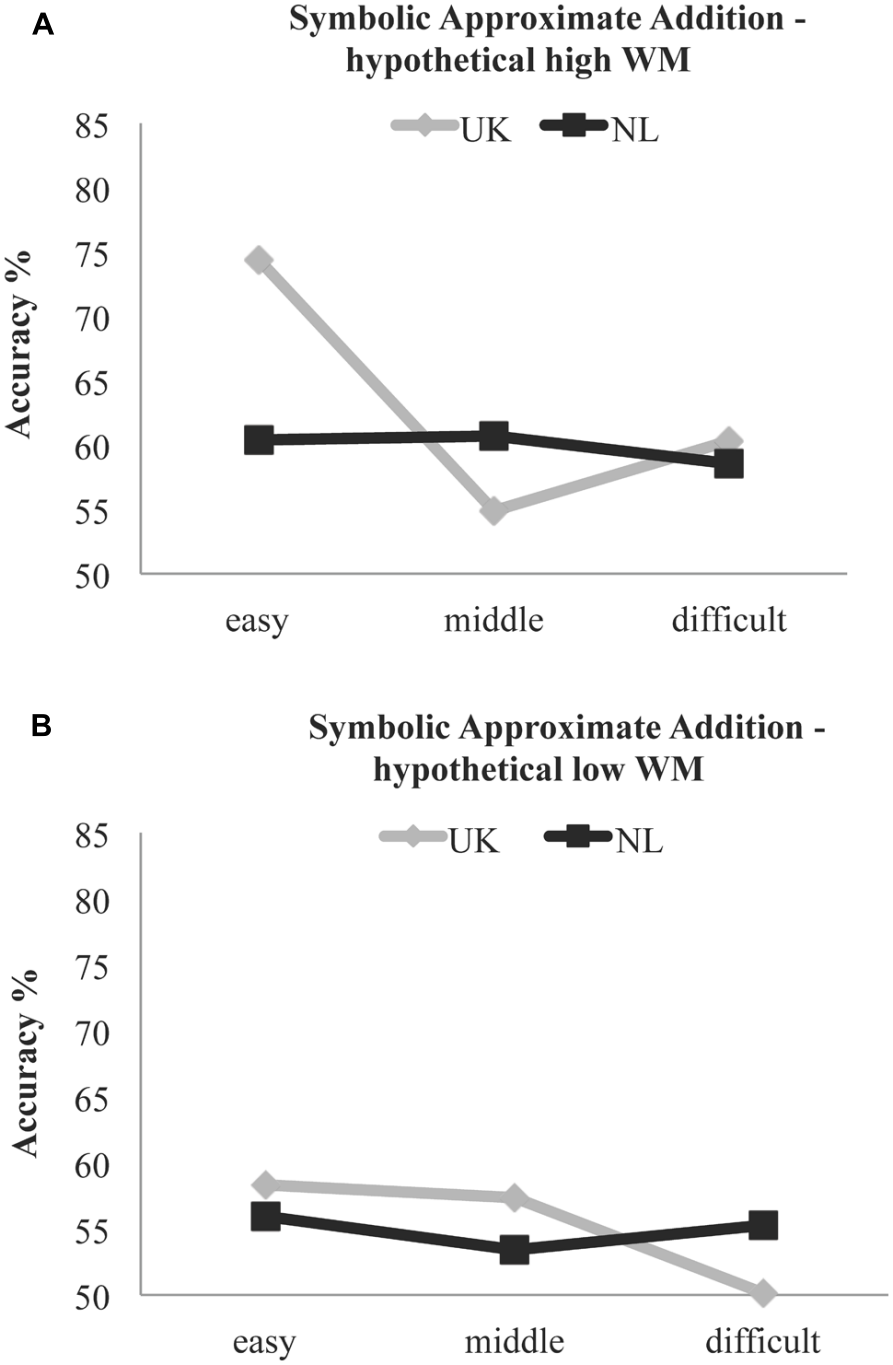
**Children’s symbolic approximate addition performance (%) using two hypothetical values in the centered CE WM measure [1 *SD* above (A) and below (B) the mean] as substitutes in the regression equations.** For the English-speaking sample (UK), with changes in CE one notices fluctuations in the symbolic approximate ratio performance. In the Dutch-speaking sample (NL), however, changes in CE capacity seem to have no effect, suggesting a WM overload.

We computed the *Y* values (% symbolic approximate addition performance in each ratio) for 1 *SD* (1.9779) above and below (-1.9711) the mean (0) of the centered CE. In the formulas, *X*_country_ is a dummy variable with the values 0 (UK) and 1 (NL). As depicted in **Figure 4**, for the English-speaking sample, as expected, one notices that with the hypothetical high or low CE value, there are pronounced fluctuations in the ratio effect. Comparing the UK children’s performance with the hypothetical high CE in **Figure 4A** and their performance with the centered (0) CE in **Figure 3B**, the regression equations suggest that the higher their WM capacity, the better their performance was; particularly on the easy ratio of the symbolic task. In the Dutch-speaking sample, however, the ratio effect line remains almost flat no matter the changes in CE values: see **Figures 3B** and **4A,B**. In other words, we see that for the Dutch-speaking children, changes in CE performance do not lead to fluctuations in ratio performance, demonstrating the hypothesized WM overload. Extra CE capacity did not appear to help the Dutch-speaking children; contrary to the English-speaking children it did not appear to facilitate their symbolic approximate addition due to the inversion effect.

#### Naming Two-Digit Numbers

To examine our third hypothesis, we had administered to the whole English-speaking sample (*n* = 52) the “Naming Large Numbers Test.” Results showed that nonsymbolic and symbolic approximate arithmetic correlated significantly *r* = 0.38, *p* = 0.005, but, as expected, the ability to name numbers above 20 correlated highly only with symbolic arithmetic *r* = 0.50, *p* < 0.001 and not nonsymbolic *r* = 0.02, *p* = 0.908. Steiger’s *Z*-test (Hoerger, 2013) indicated that these correlation coefficients between the ability to name large numbers and the nonsymbolic and symbolic arithmetic task differed significantly *Z*_H_ = 3.24, *p* = 0.001.

Accumulatively, our results indicated that number naming characteristics, such as the inversion property entailed in the Dutch number naming system could affect the onset of children’s symbolic approximate arithmetic. We demonstrated that English-speaking children perform better even at a younger age. But can Dutch children even name numbers above 20 at 5 years of age? To answer this question we administered the “Naming Large Numbers Test” to a new Dutch-speaking sample (114 children; 65 boys, *M*_age_ = 5.4 years, *SD* = 0.40) matched with the English-speaking one on age (*p* = 0.30). The English-speaking sample could name correctly significantly more two-digit numbers, *F*(1,167) = 7.70, *p* = 0.006, *M_UK_* (*SD*) = 5.63 (2.52); *M_NL_* (*SD*) = 4.34 (2.41), and faster (ms), *F*(1,154) = 135.31, *p* < 0.001, *M_UK_* (*SD*) = 2154.29 (1545.11); *M_NL_* (*SD*) = 10035.28 (4428.58), than their Dutch-speaking peers. These results showed that at 5 years of age Dutch-speaking children are able to name correctly almost half of the presented two-digit numbers but are worse compared to their English-speaking peers.

### Discussion

In this experiment, we compared English-speaking and Dutch-speaking children’s symbolic approximate arithmetic performance controlling for their nonsymbolic approximate arithmetic, simple exact addition and counting skills, as well as WM ability. Also, the two samples did not differ with respect to SES background. Results confirmed our hypotheses. We found that language, specifically differences in the transparency of the number naming system such as the inversion property, can affect the developmental onset of symbolic approximate arithmetic performance. Dutch-speaking kindergarteners lagged behind English-speaking children in symbolic approximate addition, despite being older, and indirectly demonstrated a WM overload in the ratio effect of this form of arithmetic. Furthermore, we found that the ability to name two-digit numbers, which involves the inversion property in Dutch, correlates significantly with symbolic approximation and not nonsymbolic. English-speaking children were better in naming two-digit numbers than their Dutch-speaking peers.

Contrary to Gilmore et al. (2007), who had found the characteristic ratio effect in English-speaking kindergarteners’ symbolic approximate addition, Xenidou-Dervou et al. (2013) found no ratio effect in Dutch-speaking kindergarteners’ symbolic approximate addition. It should be noted that Gilmore et al.’s (2007) study was conducted with small samples (*n* = 20) drawn from a highly educated community, whereas Xenidou-Dervou et al. (2013) assessed the approximation skills in a large sample, which included a variety of SES backgrounds. But a more pronounced sample difference between the two studies was the language used. The Dutch number naming system involves the cognitively demanding inversion property. Symbolic approximate arithmetic trials involve many two-digit numbers, which entail the inversion property. Previous studies have shown that the inversion property hinders older children’s mental number line estimation ability (Helmreich et al., 2011) but had not accounted for the children’s general ability to estimate abstract quantities. Our results replicated Gilmore et al.’s (2007) findings, namely English-speaking 5 years-old performed above chance level and demonstrated the characteristic ratio effect in symbolic approximation. Dutch-speaking kindergarteners, who did not differ with the English-speaking children on SES background and math achievement, had similar nonsymbolic approximation skills. However, as expected, the Dutch-speaking kindergarteners lagged behind the English-speaking children in symbolic approximate addition, even though they were older. Specifically, Dutch children performed worse on the easy ratio, where all test trials included a two-digit number above 20 that needs to be inversed in Dutch (see Supplementary Material). The middle and the difficult ratio of the symbolic approximate addition task were difficult for both the Dutch-speaking as well as the English-speaking children (see **Figure 3B**). In the 4:7 ratio, on the other hand, which is the easiest condition, one would expect that children would have more cognitive resources left to use more effective WM strategies. This was evident for the English-speaking children in **Figure 4A**. For the Dutch-speaking children, however, that was apparently not the case. The two-digit numbers, which need to be cognitively inversed, increased the amount of cognitive resources needed and therefore performance for the Dutch-speaking children was lower than the English-speaking children and the use of effective WM strategies was not feasible (**Figure 3B**).

Nonsymbolic (Xenidou-Dervou et al., 2013, 2014) and symbolic approximation (Caviola et al., 2012; Xenidou-Dervou et al., 2013; Cragg and Gilmore, 2014) necessitate WM resources; especially the CE component of WM as defined by the well-known multicomponent model of WM (Baddeley, 1996, 2002). We had hypothesized that the demanding inversion property would affect Dutch children’s symbolic approximation, which entails numbers that are characterized by the inversion property (Zuber et al., 2009; Pixner et al., 2011). When one hears “twenty eight” one can first estimate the position of the number “twenty” on one’s mental number line and then refine this position with the use of the “eight.” However, when saying “acht en twintig” (eight and twenty) in Dutch, no mental action can be taken with the “acht”; this has to be retained in one’s WM and recalled later updating the mental estimation of the “twintig.” The ratio effect in approximation is assumed to occur because quantities that are closer to each other have a larger representational overlap on an assumed mental number line. Indeed our results verified that the difference between Dutch- and English-speaking children in symbolic approximation – not nonsymbolic – appeared to be modified by CE capacity. Contrary to the English-speaking children, examining changes in the ratio effect of symbolic approximate addition when increasing CE capacity in the Dutch-speaking sample, one notices no differences in their ratio performance. This demonstrated a significant WM load. In other words, the English-speaking children had room for change/improvement when their CE capacity allowed it, whereas Dutch-speaking children did not. The cognitive load induced by the demanding two-digit Dutch-number naming system was too high at this young age, occupying cognitive resources, which would otherwise allow room for improvement in symbolic approximate addition. It should be noted that in this study we focused on the PL component of WM and its interaction with the CE due to the hypothesized WM load derived from the phonological representations of the numbers. It would be interesting for future studies, however, to examine also the role of the visuospatial component of WM and its interaction with the CE. Furthermore, future studies should verify our findings with more experimental manipulations in order to demonstrate the causal role of WM within this context.

Furthermore, our results demonstrated for the first time, that the ability to name two-digit numbers correlates highly with symbolic approximation and not nonsymbolic. Previous studies have indicated that the inversion property affects symbolic processing even in non-verbal tasks (Helmreich et al., 2011; Göbel et al., 2014a). It seems that the mere presentation of a number symbol activates its phonological representation in arithmetic. When symbolic approximation is being proven to be an important, consistent predictor of children’s math achievement (De Smedt et al., 2013; Xenidou-Dervou et al., 2013), we demonstrate that the ability to name large numbers plays an important role in its developmental onset. Dutch kindergarteners are significantly worse in naming such numbers compared to their English-speaking peers.

The approximate addition tasks used in our experiments entailed two-digit numerosities across all their trials (see Supplementary Material). The trial construction level in this task is stringently balanced across ratios, controlling for alternatives to approximate addition strategy usage and continuous quantity variables in the nonsymbolic condition (see Supplementary Material, also Barth et al., 2006; Gilmore et al., 2010; Xenidou-Dervou et al., 2013, 2014). The inversion effect could potentially affect at any point within an arithmetic process, for example, when merely seeing the numbers in the symbolic condition, when adding them or when comparing the sum to the target quantity. Therefore, in Experiment 2 we used again all trials in order to not disturb the controlled balanced nature of the trials and examine the differences in effect on the basis of the ratio performance. In essence, only two trials in the middle ratio and three trials in the difficult ratio included numbers that do not need to be inversed in Dutch (see Supplementary Material); both of these ratios were hard for all children (see **Figure 3B**). However, all test trials in the easy ratio included an “inversion number” and that is precisely where we found the English-speaking children to outperform the Dutch-speaking children. Our findings cumulatively provide a first indication for the negative effect that the inversion property can have on the onset of symbolic arithmetic. However, future studies should design more rigorous experiments (e.g., Göbel et al., 2014a) targeting specifically the inversion effect on symbolic approximation.

## Conclusion

Cumulatively, findings from both experiments present a clear picture about the importance of education and the language of numbers in developing symbolic arithmetic. Contrary to Gilmore et al. (2007), the present study’s results demonstrate that symbolic arithmetic *does* need instruction; it needs instruction of numbers. We showed that development and education modulate symbolic arithmetic more than the ANS. Furthermore, we demonstrated that in contrast to the ANS; symbolic processing is modulated by language. In Experiment 1, testing a large Dutch sample, we showed that nonsymbolic and symbolic approximate addition have distinct developmental trajectories, with the latter demonstrating significant growth after the start of formal schooling (primary school). In the Dutch-speaking population, symbolic approximate arithmetic onsets in grade 1, not earlier. In Experiment 2, we saw that for English-speaking children, this ability can start earlier. That is because the Dutch number naming system is cognitively more demanding: it involves the inversion property. Our findings demonstrated that Dutch-speaking kindergarteners: (1) Lagged behind English-speaking children in symbolic arithmetic, not nonsymbolic; (2) Demonstrated a WM overload in symbolic approximate arithmetic; not nonsymbolic, and (3) Were significantly worse in naming large numbers compared to their English-speaking peers. Furthermore, we showed that the ability to name large numbers correlated with symbolic, not nonsymbolic approximation. To our knowledge, this is the first evidence for the effect of the inversion property on the onset of symbolic approximation; a core system for the development of mathematical achievement (De Smedt et al., 2013; Xenidou-Dervou et al., 2013).

From a theoretical perspective, our findings demonstrate that while the ANS may be linked with symbolic numerosity processing (Feigenson et al., 2004, 2013; Libertus et al., 2011; Starr et al., 2013; Xenidou-Dervou et al., 2013; Gilmore et al., 2014), developing solid symbolic processing skills goes beyond simple ANS representations. The symbolic number system is modulated more by education and development. Also, language plays an essential role in this process to create solid representations for large exact numbers. Given the extensive research that indicates the importance of symbolic processing skills in the development of children’s math achievement (De Smedt et al., 2013; Xenidou-Dervou et al., 2013; Lyons et al., 2014), future studies should place more focus on the role that language plays in developing these skills. From an educational perspective, our results suggest that children who speak languages that entail the inversion property in their number naming system, such as Dutch, German, Arabic, and other (see Comrie, 2005; Göbel et al., 2011), should place more focus in learning and automatizing two-digit numbers since they are cognitively more demanding compared to other – more transparent – number naming systems. For Dutch-speaking children, our findings suggest that it could potentially be useful to start receiving formal school instruction on Arabic numbers already from kindergarten. There is increasing evidence in older children (Göbel et al., 2011, 2014a; Helmreich et al., 2011) and even adults (Nuerk et al., 2005) on the negative effects the inversion property can have on various mathematical abilities. As a striking example of the importance of this issue, one of our Dutch sample’s teachers reported that she overheard a child telling another in class while doing arithmetic: “Just say the numbers in English, it’s easier.” In times when the transfer of knowledge and skills is prominent and international student assessments prevail, improving early educational instruction is of primary importance.

## Conflict of Interest Statement

The authors declare that the research was conducted in the absence of any commercial or financial relationships that could be construed as a potential conflict of interest.

## Footnotes

1 http://vu.mathchild.nl/en/home/

2 www.summerscientist.org
