# Supplementary material for: The developmental onset of symbolic approximation: beyond nonsymbolic representations, the language of numbers matters
Source: Front Psychol. 2015 Apr 29;6:487. doi: 10.3389/fpsyg.2015.00487 (PMC4413728; doi:10.3389/fpsyg.2015.00487)
Supplement: Supplementary file 1 [file Data_Sheet_1.DOCX]

**Appendix**

*Test Trials of the Nonsymbolic and Symbolic Approximate Tasks from Xenidou-Dervou et al. (2013; 2014)*

|  | **Ratio** | **First blue array (B1)** | **Second blue array (B2)** | **Sum of blue** | **Red array (R)** | **Correct Response** | **Systematic strategies** ^a^ | | | | | **Continuous**  **Quantity** ^c^ |
| --- | --- | --- | --- | --- | --- | --- | --- | --- | --- | --- | --- | --- |
|  |  |  |  |  |  |  | **Near/far** | **Blue** | **Red** | **B2 vs R** | **B1 vs R** |  |
| **1** | 4:7 | 6 | 6 | 12 | 21 | Red | Far (0.29) | -1 ^b^ | 1 | 1 | 1 | B |
| **2** | 4:7 | 8 | 12 | 20 | 35 | Red | Medium (0.34) | -1 | 1 | 1 | 1 | A |
| **3** | 4:7 | 15 | 13 | 28 | 49 | Red | Far (0.31) | -1 | 1 | 1 | 1 | B |
| **4** | 4:7 | 16 | 20 | 36 | 63 | Red | Far (0.32) | -1 | 1 | 1 | 1 | A |
| **5** | 7:4 | 20 | 8 | 28 | 16 | Sum Blue | Near (0.80) | 1 | -1 | -1 | 0 | B |
| **6** | 7:4 | 30 | 12 | 42 | 24 | Sum Blue | Near (0.80) | 1 | -1 | -1 | 0 | A |
| **7** | 7:4 | 6 | 50 | 56 | 32 | Sum Blue | Medium (0.64) | 1 | -1 | 1 | -1 | B |
| **8** | 7:4 | 7 | 63 | 70 | 40 | Sum Blue | Medium (0.63) | 1 | -1 | 1 | -1 | A |
| **9** | 4:6 | 8 | 8 | 16 | 24 | Red | Far (0.33) | -1 | 1 | 1 | 1 | B |
| **10** | 4:6 | 16 | 8 | 24 | 36 | Red | Medium (0.44) | -1 | 1 | 1 | 1 | A |
| **11** | 4:6 | 12 | 20 | 32 | 48 | Red | Medium (0.42) | -1 | 1 | 1 | 1 | B |
| **12** | 4:6 | 20 | 20 | 40 | 60 | Red | Far (0.33) | -1 | 1 | 1 | 1 | A |
| **13** | 6:4 | 11 | 7 | 18 | 12 | Sum Blue | Near (0.92) | 1 | -1 | -1 | 0 | B |
| **14** | 6:4 | 33 | 7 | 42 | 28 | Sum Blue | Near (0.85) | 1 | -1 | -1 | 0 | A |
| **15** | 6:4 | 25 | 35 | 60 | 40 | Sum Blue | Near (0.88) | 1 | -1 | 0 | -1 | B |
| **16** | 6:4 | 10 | 26 | 36 | 24 | Sum Blue | Near (0.92) | 1 | -1 | 0 | -1 | A |
| **17** | 4:5 | 7 | 9 | 16 | 20 | Red | Medium (0.45) | -1 | 1 | 1 | 1 | B |
| **18** | 4:5 | 12 | 12 | 24 | 30 | Red | Medium (0.40) | -1 | 1 | 1 | 1 | A |
| **19** | 4:5 | 24 | 8 | 32 | 40 | Red | Medium (0.60) | -1 | 1 | 1 | 1 | B |
| **20** | 4:5 | 34 | 6 | 40 | 50 | Red | Near (0.68) | -1 | 1 | 1 | 1 | A |
| **21** | 5:4 | 6 | 14 | 20 | 16 | Sum Blue | Near (0.88) | 1 | -1 | 0 | -1 | B |
| **22** | 5:4 | 15 | 50 | 65 | 52 | Sum Blue | Near (0.96) | 1 | -1 | 0 | -1 | A |
| **23** | 5:4 | 32 | 8 | 40 | 32 | Sum Blue | Near (1.00) | 1 | -1 | -1 | 0 | B |
| **24** | 5:4 | 40 | 10 | 50 | 40 | Sum Blue | Near (1.00) | 1 | -1 | -1 | 0 | A |

^a^ These columns present information for the given trials with regard to the usage of possible systematic response strategies: Near/far = response based on the ratio distance between the larger blue addend and the red; Blue = only the blue response is chosen; Red = only the red response is chosen; B2 vs Rstr = only the second blue addend is compared; B1vsRstr only the first blue addend is compared,

^b^ 1 = predicts correct answer for that trial, -1: predicts a false answer for that trial, 0 = does not provide a clear prediction

^c^ Continuous quantity conditions: A = dot size, total dot surface area, total dot contour length and density positively correlated with number while array size negatively correlated with number;

B = dot size, total dot surface area, total dot contour length and density negatively correlated with number while array size positively correlated with number.
